# Dynamics of intestinal multidrug-resistant bacteria colonisation contracted by visitors to a high-endemic setting: a prospective, daily, real-time sampling study

**DOI:** 10.1016/S2666-5247(20)30224-X

**Published:** 2021-04

**Authors:** Anu Kantele, Esther Kuenzli, Steven J Dunn, David A B Dance, Paul N Newton, Viengmon Davong, Sointu Mero, Sari H Pakkanen, Andreas Neumayr, Christoph Hatz, Ann Snaith, Teemu Kallonen, Jukka Corander, Alan McNally

**Affiliations:** a Department of Infectious Diseases, University of Helsinki and Helsinki University Hospital, Helsinki, Finland; b Human Microbiome Research Program, University of Helsinki, Helsinki, Finland; c Department of Mathematics and Statistics, University of Helsinki, Helsinki, Finland; d Department of Medicine, Swiss Tropical and Public Health Institute, Basel, Switzerland; e University of Basel, Basel, Switzerland; f Department of Public and Global Health, Epidemiology, Biostatistic and Prevention Institute, University of Zurich, Zurich, Switzerland; g Institute of Microbiology and Infection, College of Medical and Dental Sciences, University of Birmingham, Birmingham, UK; h Lao-Oxford-Mahosot Hospital-Wellcome Trust Research Unit, Microbiology Laboratory, Mahosot Hospital, Rue Mahosot, Vientiane, Lao; i Centre for Tropical Medicine and Global Health, Nuffield Department of Medicine, University of Oxford, Oxford, UK; j Faculty of Infectious and Tropical Diseases, London School of Hygiene & Tropical Medicine, London UK; k Department of Infectious Diseases and Hospital Hygiene, Cantonal Hospital, St Gallen, Switzerland; l Department of Infectious Diseases and Hospital Hygiene, Cantonal Hospital, St Gallen, Switzerland; m Department of Biostatistics, Institute of Basic Medical Sciences, Faculty of Medicine, University of Oslo, Oslo, Norway; n Parasites and Microbes, Wellcome Sanger Institute, Hinxton, Cambridgeshire, UK

## Abstract

**Background:**

Antimicrobial resistance is highly prevalent in low-income and middle-income countries. International travel contributes substantially to the global spread of intestinal multidrug-resistant Gram-negative bacteria. Hundreds of millions of annual visitors to low-income and middle-income countries are all exposed to intestinal multidrug-resistant Gram-negative bacteria resulting in 30–70% of them being colonised at their return. The colonisation process in high-exposure environments is poorly documented because data have only been derived from before travel and after travel sampling. We characterised colonisation dynamics by exploring daily stool samples while visiting a low-income and middle-income countries.

**Methods:**

In this prospective, daily, real-time sampling study 20 European visitors to Laos volunteered to provide daily stool samples and completed daily questionnaires for 22 days. Samples were initially assessed at Mahosot Hospital, Vientiane, Laos, for acquisition of extended-spectrum β-lactamase-producing (ESBL) Gram-negative bacteria followed by whole-genome sequencing of isolates at MicrobesNG, University of Birmingham, Birmingham, UK. The primary outcome of the study was to obtain data on the dynamics of intestinal multidrug-resistant bacteria acquisition.

**Findings:**

Between Sept 18 and Sept 20, 2015, 23 volunteers were recruited, of whom 20 (87%) European volunteers were included in the final study population. Although colonisation rates were 70% at the end of the study, daily sampling revealed that all participants had acquired ESBL-producing Gram-negative bacteria at some point during the study period; the colonisation status varied day by day. Whole-genome sequencing analysis ascribed the transient pattern of colonisation to sequential acquisition of new strains, resulting in a loss of detectable colonisation by the initial multidrug-resistant Gram-negative strains. 19 (95%) participants acquired two to seven strains. Of the 83 unique strains identified (53 *Escherichia coli*, 10 *Klebsiella* spp, and 20 other ESBL-producing Gram-negative bacteria), some were shared by as many as four (20%) participants.

**Interpretation:**

To our knowledge, this is the first study to characterise in real-time the dynamics of acquiring multidrug-resistant Gram-negative bacterial colonisation during travel. Our data show multiple transient colonisation events indicative of constant microbial competition and suggest that travellers are exposed to a greater burden of multidrug-resistant bacteria than previously thought. The data emphasise the need for preventing travellers' diarrhoea and limiting antibiotic use, addressing the two major factors predisposing colonisation.

**Funding:**

The Finnish Governmental Subsidy for Health Science Research, The Scandinavian Society for Antimicrobial Chemotherapy, the Sigrid Jusélius Foundation, Biotechnology and Biological Sciences Research Council; Wellcome Trust, Medical Research Council; The Royal Society; Joint Programming Initiative on Antimicrobial Resistance, and European Research Council.

## Introduction

Antimicrobial resistance poses a serious threat to human health worldwide.^1^ The rapid global spread of multidrug-resistant clones of *Escherichia coli, Klebsiella pneumoniae*, and other Enterobacteriaceae raises an alarming public health concern.^1^ Worldwide dissemination of successful clones, such as *E coli* sequence type (ST)131, has been the primary driver of the increased prevalence of extended-spectrum β-lactamase (ESBL)-producing *E coli* in clinical isolates.^2^ Correspondingly, the global spread of carbapenemase-producing clones of *E coli,* such as ST410^3^ and ST167,^4^ and *K pneumoniae* clones, such as CG258 and ST11,^5^ largely account for the rapid emergence of carbapenem resistance in clinical isolates of Gram-negative pathogens worldwide.

The literature suggests international travel is strongly associated with the acquisition of multidrug-resistant Gram-negative strains; ESBL-producing *E coli* bacteria are the most common, with 30–70% of travellers colonised.^6^, ^7^, ^8^, ^9^, ^10^, ^11^ The carriage rate of intestinal multidrug-resistant Gram-negative bacteria is highest in people from the south and southeast Asia, followed by Africa and South America. People visiting these high-risk regions are at substantial risk of acquiring multidrug-resistant Gram-negative bacteria.^11^ Colonisation occurs even during short visits (1–5 days),^6^, ^7^, ^8^, ^9^, ^10^, ^11^ can last for months or even over a year without antimicrobial use,^6^, ^8^, ^9^, ^12^ and can lead to further spread after return home.^6^, ^9^ Genome-level analysis of multidrug-resistant strains colonising travellers suggests that newly acquired multidrug-resistant strains tend to displace resident intestinal commensal *E coli* strains alongside new non-multidrug-resistant strains, so that the pretravel population remains but as a minority.^13^

**Research in context**

**Evidence before this study**

We searched PubMed using the terms “beta-lactamase [MeSH]”, and “travel” from the inception of the database to Dec 31, 2019, for human studies in any language. Over the past decade, several investigations reported a high incidence of travel-associated colonisation by extended-spectrum β-lactamase (ESBL)-producing Enterobacteriaceae for travellers returning from tropical and subtropical countries. Apart from destination, travellers' diarrhoea and antibiotic use abroad were identified as major factors predisposing to colonisation. So far, studies exploring travel-acquired ESBL- producing Enterobacteriaceae have focused on findings in post-travel stool. Only one of the investigations reviewed covered the initial stages of acquisition, analysing colonisation dynamics over time, but this study did not use real-time analyses or whole-genome sequencing.

**Added value of this study**

To our knowledge, this is the first report characterising in real-time the dynamics of ESBL-producing Enterobacteriaceae colonisation during travel to low-income and middle-income countries, with daily stool specimens immediately analysed in a modern microbiology laboratory (Wellcome Trust) on location. Sampling solely after travel would have yielded a multidrug-resistant Gram-negative colonisation rate of 70%, but daily specimen collection revealed a 100% colonisation rate. The acquisition was found to be transient in nature, with detectable colonisation generally lasting one or a few days at most. By analysing isolates from selective primary stool cultures by whole-genome sequencing, the number of individual strains contracted was found to be much higher than anticipated. 19 of 20 participants acquired several different ESBL-producing Enterobacteriaceae strains (2–7 per participant), with a total of 83 individual strains. Our data show that the numerous studies of travellers' colonisation rates have, to date, underestimated the burden. When sampling only before and after travel, the actual burden of multidrug-resistant bacteria is much smaller than it really is because many episodes of multidrug-resistant Gram-negative acquisition lead to transient colonisation were no longer verifiable after travel. Revealing this transient pattern of colonisation where sequential acquisitions of new strains far exceed the figures previously reported, our data considerably widen the current understanding of multidrug-resistant acquisition abroad.

**Implications of all the evidence available**

Our novel observation of multiple transient multidrug-resistant acquisitions in visitors to low-income and middle-income countries unravels the dynamics of the colonisation event. Its transient nature implies a competition between resistant strains and an individual's own microbiota, as suggested by the generally rapid disappearance of the bacteria soon after return to a high-income country. Besides revealing the antimicrobial resistance burden abroad, the surprisingly high number of multidrug-resistant strains acquired might indicate a greater risk for travellers than previously thought because each of these strains might have the potential to transfer resistance genes to members of the individuals' microbiota. Mechanisms underlying the competition between clones should be scrutinised in more detail and methods fight off colonisation already at initial stages must be identified.

In previous studies of travellers' intestinal colonisation by multidrug-resistant Gram-negative bacteria, samples have been taken immediately before travel and upon return home.^6^, ^7^, ^8^, ^9^, ^10^ As such, the dynamics of this competitive colonisation process are unknown. Here we present a longitudinal study done in Laos; the aim of the study was to characterise the colonisation dynamics using fine-scale genomic analysis of daily stool samples from volunteers.

## Methods

### Study design and participants

To obtain data on the dynamics of intestinal multidrug-resistant bacteria acquisition, we characterised the colonisation process by ESBL-producing Gram-negative bacteria by sampling participants' stools on arrival in Vientiane, Laos, each day abroad, and at departure. The study was done over 22 days.

Volunteers were recruited prospectively from participants at a medical course held from Sept 19 to Oct 9, 2015, in Vientiane. We also invited the recruitees' companions to volunteer. Each participant was asked to provide daily stool samples while staying in Vientiane. Those collected within the first 2 days were considered as baseline samples and the final stool sample was considered the departure samples. Questionnaires assessing background information and travel-related data were used at recruitment and before departure. During the stay, the volunteers were asked to complete a health card to record gastrointestinal symptoms, food habits, and medication use each day. Travellers' diarrhoea was defined as passage of three or more loose or liquid stools per day. Any ESBL-producing Gram-negative bacteria not found in the baseline samples but detected in one or more stool samples taken later were defined as travel-acquired ESBL-producing Gram-negative bacteria. Only volunteers providing at least five daily samples over the 22-day period were included in the final cohort. The study protocol (appendix 1) was approved by the Ethics Committee of the Helsinki University Hospital, Helsinki, Finland, and the Ethics Committee of Northwest and Central Switzerland, Basel, Switzerland. All participants provided written informed consent.

### Procedures

The initial screening for presumptive ESBL-producing Gram-negative bacteria strains from stool samples was done at the Microbiology Laboratory of Mahosot Hospital, Vientiane, Laos by culture on CHROMagar ESBL agar plates (CHROMagar, Paris, France). Phenotypically distinct colonies were subcultured and stored at −80°C in Protect tubes (Technical Service Consultants, Heywood, UK) with the original swabs. The samples were then transported by plane on dry ice first to the Swiss Tropical and Public Health Institute, Basel, Switzerland, and then to the University of Helsinki, Helsinki, Finland, for analyses. In Finland, the bacteria were recultured on an equivalent chromogenic media, ChromID ESBL agar (BioMérieux, Marcy-l'Étoile, France), and a colony of all morphotypes present on the ChromID plate was subcultured and cryopreserved. The isolates were shipped on dry ice to the University of Birmingham, Birmingham, UK, for genome sequencing by the MicrobesNG facility. Libraries were prepared using the Nextera XT kit (Illumina, San Diego, CA, USA) and sequenced on the Illumina HiSeq platform (Illumina).

Illumina genome sequence reads were assessed for quality using FastQC (version 0.11.9) and subsequently trimmed using Trimmomatic (version 0.3),^14^ with a sliding window quality of Q15 and length of 20 base pairs. Kraken (version 2) was used to speciate isolates against all bacterial, archaeal, and viral genomes within the RefSeq database up to Nov 1, 2017. De novo assembled genomes were produced using SPAdes (version 3.13.0) under default conditions, with the inclusion of the careful flag.^15^ Assemblies were also built using SKESA (version 2.3.0) under default conditions. Resulting assembled genomes were annotated using Prokka (version 1.11) under default conditions.^16^

Antibiotic resistance genes were detected in assembled and annotated genomes using Abricate (version 0.8.7) and the ResFinder database. Abricate detected all genes with a minimum identity of 75% and a minimum coverage of 0%. Partial hits to genes were then manually assessed because of the occasional splitting of genes (particularly on plasmids). Genes that were present in the total assembly at a coverage of 90% were determined to be present. Prokka-annotated genomes were manually inspected to confirm the presence of resistance genes identified. MLST (version 2.15) was used to verify species identification and assign classical ST designations to isolates. Phylogroups were assigned using ClermonTyping.

Isolates were examined for potential relationships by criteria including participant number, ST, resistance profile, and phylogenetic distribution. Where isolates were suspected of sharing recent source or transmission events, Snippy (version 4.3.6) was used to map reads of isolates against the assembled genome of the earliest relative isolate. The assemblies of all isolates within identified clusters were highly similar (appendix 2 p 7). The number of single nucleotide polymorphisms between strains was determined using snp-dists (version 0.6.3).

Phylogenies were reconstructed using RaxML-NG under the GTR-GAMMA model (version 0.6.0), a core single nucleotide polymorphism alignment from Snippy-core (version 4.3.6). Phylogenies were midpoint rooted and combined with metadata for visualisation in Phandango (version 1.3.0). *E coli* isolates were screened for virulence factors using the ecoli_vf and vfdb databases bundled in the base version of Abricate. These isolates were then assigned to pathotypes using a previously described scheme^17^

### Outcomes

The primary outcome of the study is real-time data on the dynamics of intestinal multidrug-resistant bacteria acquisition while visiting low-income and middle-income countries

### Role of the funding source

The funders had no role in study design, data collection, data analysis, data interpretation, or writing of this Article.

## Results

**Figure 1:**
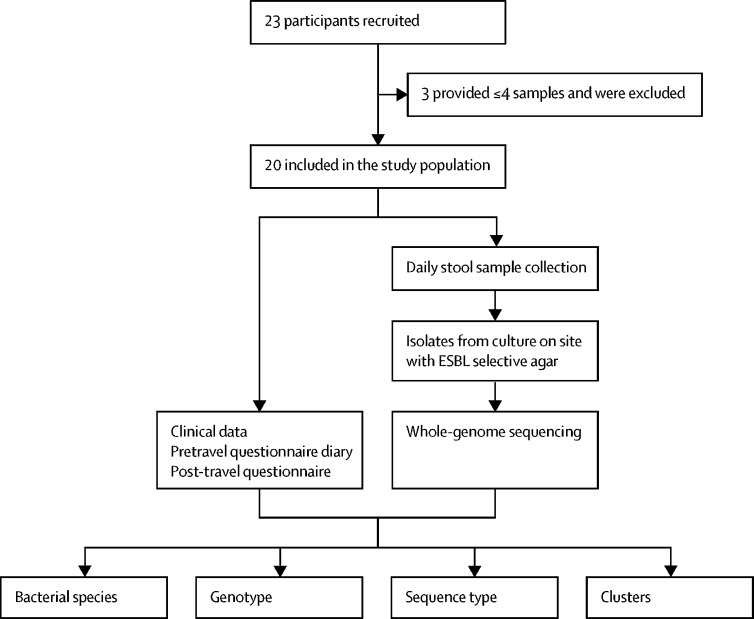
Study design ESBL=extended-spectrum β-lactamase.

Between Sept 18 and Sept 20, 2015, a total of 23 volunteers were recruited (appendix 2 p 2), three of whom were excluded because they only provided two samples (of note, ESBL Gram-negative bacteria was found in all six samples). 20 (87%) European volunteers were included in the final study population (figure 1). Of the volunteers, ten (50%) were younger than 50 years, 11 (55%) were women, and 19 (95%) were medical doctors. The median age was 42·5 years (IQR 33·5−57·0), and the median duration of stay in Laos was 20 days (IQR 12−21). Five (25%) participants had used antimicrobial medication during the previous year, three (15%) arrived directly from another tropical region, one (5%) had visited the tropics within the past 3 months, and seven (35%) had visited the tropics within the last year (appendix 2 p 2). The group provided a total of 236 stool samples.

During the sampling period, the volunteers stayed at three separate hotels, visited various restaurants either in small groups or all together, and participated in daily rounds at local hospitals. Four (20%) participants reported travellers' diarrhoea and one (5%) took antibiotics (appendix 2 p 2).

**Figure 2:**
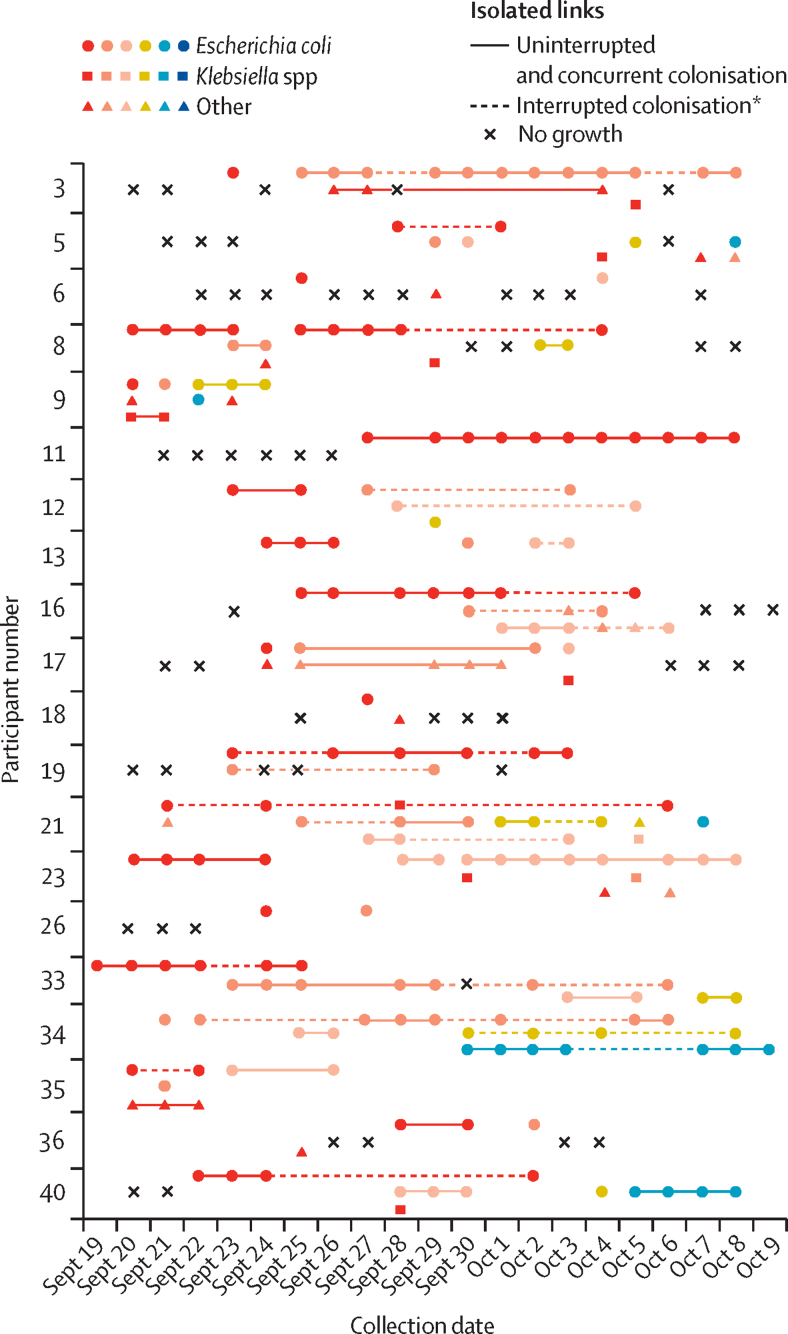
Colonisation of participants by ESBL-producing Gram-negative bacteria Strains are shown by participant, with a maximum of five differing strains identified from the samples of a single individual. Because of the large number of constituent strains in the database, the colour designations do not represent the same strains across multiple volunteers. Days where no symbol is present indicates that a participant was unable to produce a sample. ESBL=extended-spectrum β-lactamase. *Dashed lines represent maintenance of a single strain over multiple days interrupted by colonisation by another strain of the same species or a period of no growth.

ESBL-producing Gram-negative bacteria strains were detected in the primary samples of seven (35%) participants, including three who had arrived in Laos directly from another Asian country. For all participants at least one stool specimen tested positive for ESBL-producing Gram-negative bacteria in culture by day 10 of their visit (figure 2). Of the 236 faecal samples collected, 174 (74%) contained detectable ESBL-producing Gram-negative bacteria, yielding a total of 306 isolates. Of these, 292 (95%) isolates were successfully whole-genome sequenced. *E coli* was the most abundant species isolated, accounting for 219 (75%) of the 292 isolates. The other species identified were *Citrobacter* spp (28 [10%] isolates), *Klebsiella* spp (16 [5%] isolates), *Acinetobacter* spp (12 [4%] isolates), and *Enterobacter cloacae* (11 [4%] isolates); a number of other low-prevalence species, including *Aeromonas* spp and *Stenotrophomonas maltophilia* were also present (appendix 2 pp 18−28). When allocating individual isolates to the participants, colonisation by any given ESBL-producing Gram-negative bacteria during the study was clearly transient in nature: isolates were detected in only one or a few samples obtained from any given individual, sometimes with days between isolation.

**Figure 3:**
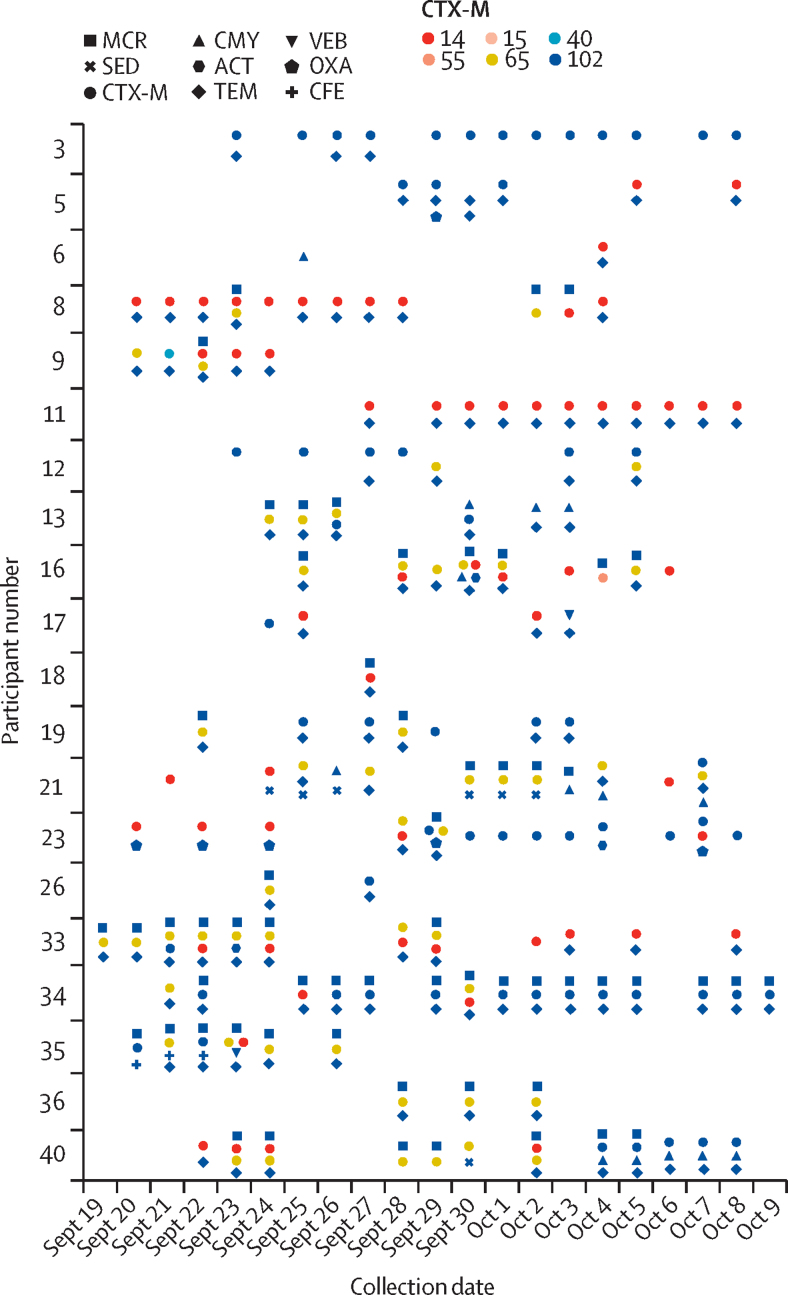
Resistance determinants identified in ESBL-producing Gram-negative bacteria isolates The different CTX-M subtypes are represented by the different coloured circles.

The genomes of all 292 isolates were found to contain at least one antimicrobial resistance gene. The most prevalent ESBL gene type was *bla*_CTX-M_found in a total of 226 (77%) isolates, with CTX-M-55 (64 [28%]), CTX-M-14 (58 [26%]), CTX-M-159 (57 [25%]), CTX-M-15 (30 [13%]), and CTX-M-102 (25 [11%]) the most common types (appendix 2 p 6). Some isolates contained multiple CTX-M types. Mobile colistin resistance genes were found in 82 isolates (28%), all but two of which were *E coli* (one *Aeromonas* sp and one other *K pneumoniae*). Superimposing MLST (multilocus sequence typing) designation (figure 2; appendix 2 p 4) and *bla/*mobile colistin resistance gene type (figure 3) onto each participants isolates provided further insight into the transient nature of gut colonisation (figure 3). Although one (5%) participant was found to have contracted a single ST2067 strain of *E coli* carrying CTX-M-15, the other 19 (95%) participants were colonised by multiple *E coli* STs and co-colonised by other ESBL-producing Gram-negative bacteria species. For example, one participant took azithromycin for travellers' diarrhoea from Sept 21 to 23, 2015, and was transiently colonised by five different *E coli* strains during the study, each with different *bla* gene repertoires. Participant 16 had a regular flux between isolation of an ST38, ST93, and ST101 strain, whereas participant 40 had initial colonisation by an ST48 strain, which was later displaced by an ST38 strain. All these strains have unique signatures of carriage of multiple *bla* genes, suggesting that travellers are exposed to a large number of multidrug-resistant Gram-negative bacteria and multidrug-resistance conferring genes during the initial colonisation process (figure 3). 19 (95%) participants acquired between two and seven strains. All the strains were identified as extraintestinal pathogenic *E coli*, with the exception of a small number of strains (seven [3%] of 219 *E coli* isolates) containing a Virulence associated gene profile and ST designation consistent with multidrug-resistant atypical enteropathogenic *E coli* strains (appendix 2 pp 9−17).

**Figure 4:**
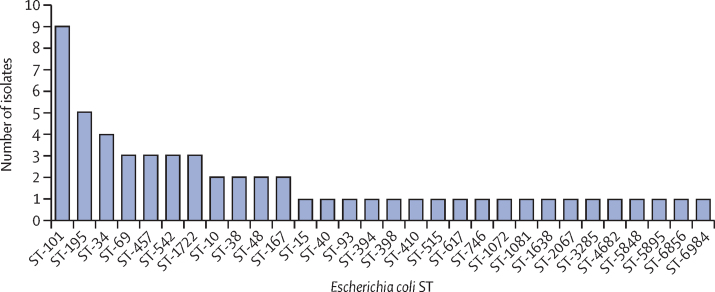
Abundance of unique STs in the stool samples of volunteers Isolates were examined using a high-resolution single nucleotide polymorphism analysis. Isolates belonging to the same sequence type were genomically deduplicated to avoid a single persistent strain biasing the results. ST=sequence type.

We analysed the population of *E coli* isolates at MLST designation level (figure 4). The most common *E coli* STs identified were ST101, ST34, ST38, and ST195. These lineages are very uncommon in surveys of ESBL resistant and carbapenem resistant *E coli*, both in isolates from Europe and previous human isolates from Laos.^18^, ^19^ Superimposing the presence of specific ESBL-producing *E coli* and mobile colistin resistance genes onto a phylogenetic tree of the *E coli* isolates revealed *bla*_CTX-M_ genes to be ubiquitous throughout the sampled population, with mobile colistin resistance genes also widely distributed across the population (appendix 2 p 5). Analysis of the *K pneumoniae* lineages showed isolates belonging predominantly to ST2176 and ST37, neither of which are well characterised, globally disseminated clones.^20^

**Figure 5:**
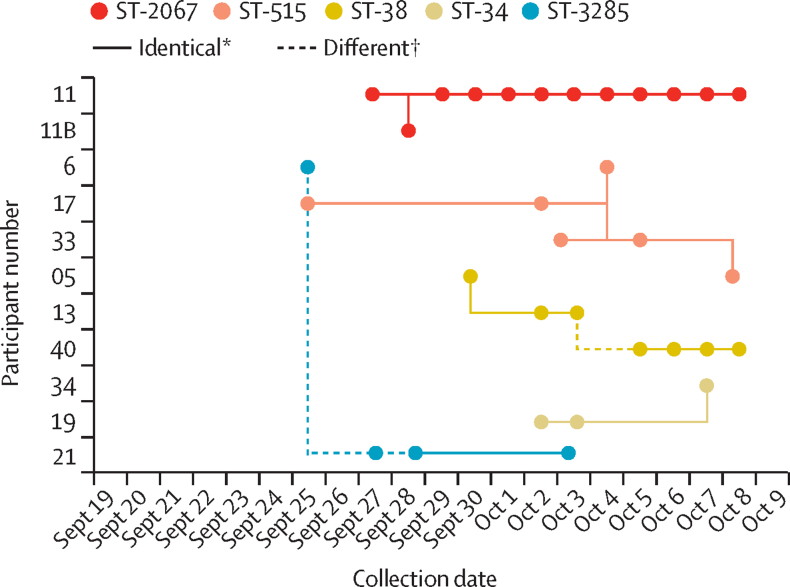
Linkage of isolates between participants High resolution SNP analysis identified several instances of a single strain colonising multiple participants. Most strains did not contain any variation. 5 of the 33 isolates contained between 1-5 SNPs. The most prolifically shared strain (ST-515) found in participants 5, 6, 17, and 33. Participant 11B is a contact of participant 11, who was found to be colonised by a shared strain. SNP=single nucleotide polymorphism. ST=sequence type. *Isolates that were identical, with no SNPs. †Isolates contained 1–5 SNPs.

When analysing the population structure of the *E coli* isolates, very little diversity was found in strains within each of the different lineages. To investigate relationships between isolates within the lineages, a high-resolution single nucleotide polymorphism analysis using the first isolated strain as a reference was done. Our data showed common strains colonising participants, often with no single nucleotide polymorphisms difference between them (figure 5). An identical ST515 strain colonised participants 6, 17, 33, and 5, and an identical ST38 colonised participants 5, 40, and 13. Participants 19 and 34 shared an identical ST34 strain, and participants 6 and 21 shared an identical ST385 strain. Participant 11 was colonised on day 8 by an ST2067 isolate that was also isolated from their companion (particpant 11B, who only provided one sample) the following day (figure 5).

## Discussion

By combining personal data of 20 European visitors to Laos with fine-scale genomic analyses of the strains isolated from their daily faecal samples, we showed that colonisation by an ESBL-producing Gram-negative bacteria during travel to endemic regions is an extremely dynamic process. We identified a constant influx of newly acquired ESBL-producing Gram-negative bacteria in all but one of the 20 participants. Over the duration of their visit, the volunteers were colonised by up to seven different strains, and often acquired multiple ESBL-producing Gram-negative bacteria species. Few studies have employed genome-level analyses,^13^ but several have reported isolating more than one new colonising ESBL-producing Gram-negative bacteria strain from post-travel samples.^7^, ^8^, ^9^, ^10^ Our data reveal the true scale and complexity at which drug-resistant bacteria colonise the intestinal tract in high-endemic settings; colonisation has been seriously underestimated, both with respect to rates of volunteers acquiring multidrug-resistant Gram-negative bacteria and number of individual strains contracted. In addition, our data suggest that several of our participants lost some of their travel-acquired ESBL-producing Gram-negative bacteria strains while still abroad. This indicates that previous studies solely using before and after travel sampling have under-reported the extent to which individuals are colonised by ESBL-producing Gram-negative bacteria. The result is especially relevant if we assume that multidrug-resistant Gram-negative bacteria simply drop below detection threshold, but do not vanish completely and will resurface once selection pressure increases again. There is a potential caveat to our study in that the apparent cyclic disappearance and reappearance of strains might be attributed to the sensitivity of the culture methods used, and colonising strains might have been missed when picking colonies for sequencing. Nevertheless, even this culture approach was sufficient to show the highly dynamic nature of the process, with acquisition rates far exceeding those previously reported.^7^, ^8^, ^9^

Our fine-scale genomic analysis enabled identification of a number of strains shared by the participants. Some of the strains colonised up to four (20%) participants, with between zero and five single nucleotide polymorphisms between shared strains. Although direct transmission cannot be confirmed, the clonality of the isolates suggests that the two colonisation events did not result from exposure to a common environmental reservoir. Such reservoirs are generally colonised by bacteria for extended periods of time, which leads to extensive diversity within the bacterial population.^21^ Thus, direct transmission or acquisition through common exposure, such as consumption of food or water, appears the most likely explanation.

The population of ESBL-producing *E coli* isolates in this study has an unexpected composition. Epidemiological surveys of multidrug-resistant *E coli*, especially those focusing on ESBL strains, are dominated by *E coli* ST131.^22^ Epidemiological investigations done on *E coli* in Laos have also shown ST131 to be the dominant drug-resistant lineage in the country.^18^, ^19^ However, no ST131 strains were isolated, and ST38 was the only lineage in our study that has been reported in previous investigation done in Laos.^18^, ^19^ Examining both intestinal colonisation isolates^19^ and clinical blood stream isolates,^18^ they suggested that the ESBL-*E coli* population in Laos might be particularly dynamic and prone to frequent fluctuation. Of note, ST101, ST34, and ST195, and other lineages frequently isolated in our study have never been reported as clinical ESBL-producing *E coli* isolates in other countries, including the UK where high-quality longitudinal data are available. Some reports describe ESBL-producing *E coli* ST101,^23^ but none report ST34 or ST195. *E coli* ST38 has been extensively reported as an ESBL-producing strain isolated both from humans and animals.^24^, ^25^ Of note, the data show that most *E coli* strains were extraintestinal pathogenic (enteropathogenic *E coli* strains), indicating their potential role as pathogens.

Most previous studies of travellers have only described acquisition of ESBL-producing *E coli.* A few studies have reported multiple findings of ESBL-producing diarrhoeagenic *E coli*^17^ or single findings of ESBL-producing *Klebsiella*^7^, ^10^ or carbapenemase-producing *E coli.*^8^, ^9^ Our data show that in addition to ESBL-producing *E coli* (219 [75%] of 292 strains) a substantial number of ESBL-producing non-*E coli* Gram-negative bacteria, such as *Citrobacter* (9%), *Klebsiella* (5%), *Acinetobacter* (4%), and *Enterobacter cloacae* (4%) and even low numbers of *Aeromonas spp* and *Stenotrophomonas maltophilia* were identified. This variety in the acquired strains might be ascribed to the rate of exposure to various multidrug-resistant Gram-negative bacteria because 19 participants attended a course of tropical medicine, which included daily clinical rounds at local hospitals. High multidrug-resistant Gram-negative colonisation rates have been reported in visitors hospitalised in the tropics.^26^

The complete absence of carbapenemase-producing *E coli* in these samples is also of note. We screened for ESBL-producing strains, but carbapenemase producers would also have grown on the selective plates used. Additionally, whole-genome sequencing analysis showed a complete absence of carbapenemase genes. This finding was somewhat surprising in southeast Asia where the prevalence of carbapenem-resistant Enterobacteriaceae is increasing.^27^ Such isolates were first reported in Laos in 2015,^5^ suggesting that carbapenem resistance has not yet become a major problem in the country. Additionally, other studies have shown a low carriage rate of carbapenemase genes.^28^

Even more striking were the extremely high concentrations of the mobile colistin resistance gene in our *E coli* isolates. The ST101 lineage—which dominated our isolate collection—has been identified as a driving lineage in the emergence of ESBL-producing *E coli* and mobile colistin resistance-positive *E coli* in the south and southeast Asia,^29^ but we observed mobile colistin resistance genes across a large number of lineages, which might be of substantial importance because mobile colistin resistance gene is rare in *E coli* linages surveyed to date. This confirms earlier reports showing that travel exacerbates the global spread of ESBL genes and *E coli* strains carrying mobile colistin resistance genes.^30^

Exposure at hospitals can be considered a limitation of the study because exposure to multidrug-resistant strains is more common in this setting. However, the main aim of the study was to explore the process of colonisation in a high-exposure setting. Although hospital visits might have increased the acquisition rates transient acquisition of multiple strains was also seen for two participants not visiting hospitals, and confirmed in our later studies of regular travellers over a 12-day trip to Benin (Kantele, unpublished data).

By combining real-time sampling of travellers with genome-level analyses, we have shown that colonisation by ESBL-producing Gram-negative bacteria in a high-exposure setting is an extremely dynamic process characterised by competition between resistant strains and an individual's own microbiota. The data suggest that some strains can colonise multiple participants potentially through direct transmission or acquisition from a common source. The challenge now lies in unravelling the mechanisms that underlie this process and competition between the clones, and finding tools to prevent colonisation already at its initial stages.

## Data sharing

Raw sequence data for all isolates is available via National Center for Biotechnology Information under the Bioproject accession number PRJNA558187.
