# Supplementary material for: Dynamics of intestinal multidrug-resistant bacteria colonisation contracted by visitors to a high-endemic setting: a prospective, daily, real-time sampling study
Source: Lancet Microbe. 2021 Apr;2(4):e151–8. doi: 10.1016/S2666-5247(20)30224-X (PMC8009952; doi:10.1016/S2666-5247(20)30224-X)
Supplement: Supplementary appendix 1 [file mmc1.pdf]

THE LANCET
Microbe

Supplementary appendix 1

This appendix formed part of the original submission and has been peer reviewed. We post it as supplied by the authors.

Supplement to: Kantele A, Kuenzli E, Dunn SJ, et al. Dynamics of intestinal multidrug-resistant bacteria colonisation contracted by visitors to a high-endemic setting: a prospective, daily, real-time sampling study. *Lancet Microbe* 2021; published online Feb 23. https://doi.org/10.1016/S2666-5247(20)30224-X.

Amendment to a travellers' antimicrobial resistance study

Colonization dynamics of extended-spectrum beta-lactamase-producing *Enterobacteriaceae* in travellers returning from high-endemic countries

Background and Rationale

Travel to high-endemic countries involves a considerable risk of becoming colonized by extended-spectrum beta-lactamase (ESBL)-producing *Enterobacteriaceae* (1-4). While abroad, the major factors predisposing to colonization are travellers' diarrhoea (TD) (3, 4) and antibiotic use (3, 4). It is not been known, however, how soon colonization occurs after travellers arrive in a high-endemic region. Nor has research addressed the possibility that some strains might only colonize the intestine temporarily during the travellers' stay abroad and therefore not be found any longer after their return home. Data on such transient colonization would support the idea of a continuous exposure in an environment.

A better understanding of the initial steps of colonization while abroad is fundamental for devising and implementing measures to prevent colonization among travellers.

Hypotheses: Colonization by ESBL-producing *Enterobacteriaceae* occurs within a few days (less than a week) after arriving in a high-endemic environment. There are changes among the colonizing strains over the stay, suggesting constant exposure to new strains.

Aim: To improve understanding of the dynamics of ESBL-PE colonization among travellers to high-endemic regions.

Study endpoints:

- Colonization rate with regard to time after arrival in a high-endemic area
- Detailed analysis of colonizing strains by whole-genome sequencing

Study design: Observational cohort study

Inclusion/Exclusion Criteria: Volunteers from low-endemic countries participating in a medical course in a high-endemic region.

Study Plan: Upon arrival in the high-endemic area, participants in the medical course "XXX" will be informed about the study. Written informed consent will be obtained from all volunteers. After completing a background questionnaire, the participant are requested every day to provide a rectal swab and fill in a short diary covering their symptoms, medications and behaviour. The information entered will be used to identify potential risk factors of colonization.

Statistical Analysis: The incidence of becoming colonized by multidrug-resistant enteric pathogens and the associated 95% confidence interval will be calculated.

Microbiological Analysis: While abroad, the participants' faecal samples will be screened in real time for ESBL-PE using a selective chromogenic medium. Potential isolates will be further sub-cultured and their susceptibility tested by the Vitek-2® system. The phenotypic resistance will be determined by Etest®, double disk synergy testing, and a modified Hodge test to detect ESBL- or carbapenemase-producing organisms. After primary cultures in the high-endemic region, the isolates will be shipped in dry ice by courier services over to Switzerland/Finland for further characterizations. Whole-genome sequencing will be conducted by collaborators in Norway/the UK.

Rationale for Participant Number: As this is an observational study with only a limited number of people meeting the inclusion criteria, no sample size calculation will be done.

Risks: The study does not put the participants at any risk, since no interventions will be included. All personal data will be pseudonymized already at samples collection and not made accessible to the laboratories handling the specimens. No drugs will be requested to be taken and no invasive techniques will be applied.

References:

1. Paltansing S, Vlot JA, Kraakman ME, Mesman R, Bruijning ML, Bernards AT, et al. Extended-spectrum beta-lactamase-producing enterobacteriaceae among travelers from the Netherlands. Emerging infectious diseases. 2013;19(8):1206-13.
2. Kuenzli E, Jaeger VK, Frei R, Neumayr A, DeCrom S, Haller S, et al. High colonization rates of extended-spectrum beta-lactamase (ESBL)-producing Escherichia coli in Swiss travellers to South Asia- a prospective observational multicentre cohort study looking at epidemiology, microbiology and risk factors. BMC infectious diseases. 2014;14:528.
3. Kantele A, Laaveri T, Mero S, Vilkman K, Pakkanen SH, Ollgren J, et al. Antimicrobials increase travelers' risk of colonization by extended-spectrum betalactamase-producing Enterobacteriaceae. Clinical infectious diseases : an official publication of the Infectious Diseases Society of America. 2015;60(6):837-46.
4. Lubbert C, Straube L, Stein C, Makarewicz O, Schubert S, Mossner J, et al. Colonization with extended-spectrum beta-lactamase-producing and carbapenemase-producing Enterobacteriaceae in international travelers returning to Germany. International journal of medical microbiology : IJMM. 2015;305(1):148-56.
