# Supplementary material for: Dynamics of intestinal multidrug-resistant bacteria colonisation contracted by visitors to a high-endemic setting: a prospective, daily, real-time sampling study
Source: Lancet Microbe. 2021 Apr;2(4):e151–8. doi: 10.1016/S2666-5247(20)30224-X (PMC8009952; doi:10.1016/S2666-5247(20)30224-X)
Supplement: Supplementary appendix 2 [file mmc2.pdf]

THE LANCET
Microbe

Supplementary appendix 2

This appendix formed part of the original submission and has been peer reviewed. We post it as supplied by the authors.

Supplement to: Kantele A, Kuenzli E, Dunn SJ, et al. Dynamics of intestinal multidrug-resistant bacteria colonisation contracted by visitors to a high-endemic setting: a prospective, daily, real-time sampling study. *Lancet Microbe* 2021; published online Feb 23. https://doi.org/10.1016/S2666-5247(20)30224-X.

Supplementary Methods

Illumina genome sequence reads were assessed for quality using FastQC (V 0.11.9), and subsequently trimmed using Trimmomatic (V 0.3)[19] with a sliding window quality of Q15 and length of 20 base pairs. Kraken (V 2) was used to speciate isolates against a database comprising of all bacterial, archaeal and viral genomes within refseq up to November 2017. *De novo* assembled genomes were produced using SPAdes (V 3.13.0) under default conditions, with the inclusion of the '--careful' flag.[20] Assemblies were also constructed using SKESA (V 2.3.0) under default conditions. Resulting assembled genomes were annotated using Prokka (V 1.11) under default conditions.[21]

Antibiotic resistance genes were detected in assembled and annotated genomes using Abricate (V 0.8.7, https://github.com/tseemann/abricate) and the Resfinder database. Abricate detected all genes with a minimum identity of 75% and a minimum coverage of 0%. Partial hits to genes were then manually assessed, due to the occasional splitting of genes (particularly on plasmids). Genes that were present in the total assembly at a coverage of 90% were determined to be present. Prokka-annotated genomes were manually inspected to confirm the presence of resistance genes identified. MLST (V 2.15, https://github.com/tseemann/mlst) was used to verify species identification and assign classical sequence type designations to isolates. A total of 219 *E. coli* isolates were detected, with an average of 203 contigs, total length 5,261,590 bp, GC 50.45% and N50 150,180 bp. Phylogroups were assigned using ClermonTyping.

Isolates were examined for potential relationships by criteria such as participant number, sequence type, resistance profile, and phylogenetic distribution. Where isolates were suspected of sharing recent source or transmission events, Snippy (V 4.3.6, https://github.com/tseemann/snippy) was used to map reads of isolates against the assembled genome of the earliest relative isolate. The assemblies of all isolates within identified clusters were highly similar (Table S4). The number of SNPs between strains was determined using snp-dists (V 0.6.3, https://github.com/tseemann/snp-dists).

Phylogenies were reconstructed using RaxML-NG under the GTR-GAMMA model (V 0.6.0) a core SNP alignment from Snippy-core (V 4.3.6). Phylogenies were midpoint rooted, and combined with metadata for visualisation in Phandango (V 1.3.0).

Table S1. Demographics of participants in our study exploring acquisition of extended-spectrum beta-lactamase-producing E. coli by daily stool sampling over their visit to Lao People’s Democratic Republic in September–October, 2015. Five (25%) participants [9, 11, 18, 23, 34] had used antimicrobial medication during the previous year, three (15%, [[9, 23, 34]) arrived directly from another tropical region, one (5%,[12]) had visited the tropics within the past 3 months, and seven (35%[3, 6, 8, 11, 18, 35, 36]).

ID	Age (yrs)	Sex	**Country of origin**	**Arriving from** (if not country of origin)	**Date of arrival**	**Departure date**	**Travelers' diarrhoea**	**Antibiotic use**
3	33	male	Germany		20 Sep	10 Oct	4 Oct	
5	67	male	Switzerland		20 Sep	09 Oct		
6	52	male	Finland		20 Sep	10 Oct	28 Sep	
8	29	female	Switzerland		20 Sep	09 Oct		
9	30	female	Austria	Vietnam	20 Sep	26 Sept		
11	61	female	Finland		20 Sep	10 Oct	25/26 Sep	
12	64	male	Austria		10 Sep	09 Oct		
13	38	female	Switzerland		20 Sep	10 Oct		
16	62	female	Switzerland		19 Sep	17 Oct		
17	53	female	Finland	USA	21 Sep	10 Oct		
18	46	male	Germany		25 Sep	04 Oct		
19	34	female	Netherlands		20 Sep	03 Oct		
21	53	female	Norway		20 Sep	07 Oct		
23	32	male	Austria	Vietnam	20 Sep	09 Oct		
26	20	female	Switzerland		19 Sep	28 Sep		
33	39	male	Germany		13 Sep	24 Oct		
34	35	male	Switzerland	Thailand	20 Sep	10 Oct	20 Sep–7 Oct	21–23 Sep
35	63	male	Switzerland		19 Sep	28 Sep		
36	52	female	Germany		25 Sep	04 Oct		
40	37	female	Germany		19 Sep	14 Oct		

Table S2 – Frequencies of ESBL positive gram negative taxa observed in the dataset. *E. coli* and other Enterobacteriaceae (e.g. *Citrobacter, Klebsiella*) were the most common species observed. Some genera were isolated in very low numbers, (e.g. *Stenotrophomonas, Aeromonas).*

Taxa	Number of Isolates
E. coli	219
Citrobacter	28
Klebsiella	16
Enterobacter cloacae	11
Acinetobacter	12
Other	20
Total	306

Figure S1 – Distribution of observed *E. coli* sequence types sorted by participant number and date. In some instances, there are clear single sequence types that longitudinally colonise a single participant (e.g. 1722, Participant 03). Other participants exhibit transient colonisation by multiple sequence types (e.g. Participant 33).

Participant Number

Collection Date	03	04	05	06	08	09	11	12	13	16	17	18	19	21	23	26	33	34	35	36	40
19-Sep																	34				
20-Sep		101			69	4682									5895		34		167		
21-Sep					69	1072								457	5895		34	1081	5848		
22-Sep					69	48 & 101									5895			167	167		195
23-Sep	195	101			69 & 195	48		394					101 & 34				34		34 & 101		195
24-Sep					195	48			101		1638			457	5895	10	34 & 195				195
25-Sep	1722			3285	69			394	101	101	515			101			34 & 195	617			
26-Sep	1722				69				101	101			34					617	101		
27-Sep	1722				69		2067	69				410		3285		34		167			
28-Sep			1722		69			746		101			34	3285 & 101	542		34 & 195	167		101	48
29-Sep	1722		6856				2067	101		101			101		542		34 & 195	167			48
30-Sep	1722		38				2067		1722	38 & 101			34	101	457			34		101	48
01-Oct	1722		1722				2067			93 & 101				10	457			167			
02-Oct	1722				6984		2067		38	93	515		34	10	457		195	34 & 195		398	195
03-Oct	1722				6984		2067		38	93	542		34	3285	457		515	34			
04-Oct	1722			515	69		2067			38				10	457			34 & 195			40
05-Oct	1722		542				2067			101							515	167			38
06-Oct							2067			93				457	457			167			38
07-Oct	1722						2067							69	457		195	34			38
08-Oct	1722		515				2067								457		38	34 & 195			38
09-Oct																		34			

Figure S2 – Phylogeny of *E. coli* isolates shown with presence of observed beta-lactamase genes. The majority of isolates carried at least one type of CTX-M, and an alarming amount of isolates also carried colistin resistance gene MCR. Some less common beta-lactamase genes were also observed (e.g. ACT, ADC). Purple = gene present, orange = gene absent. Phylogeny reconstructed using RaxML-NG, based on a Snippy core SNP alignment, and visualised in Phandango. The tree uses reference isolate CFT073, highlighted in green. The tree is midpoint rooted. Number of SNP sites = 2405. MCR = Mobile colistin resistance.

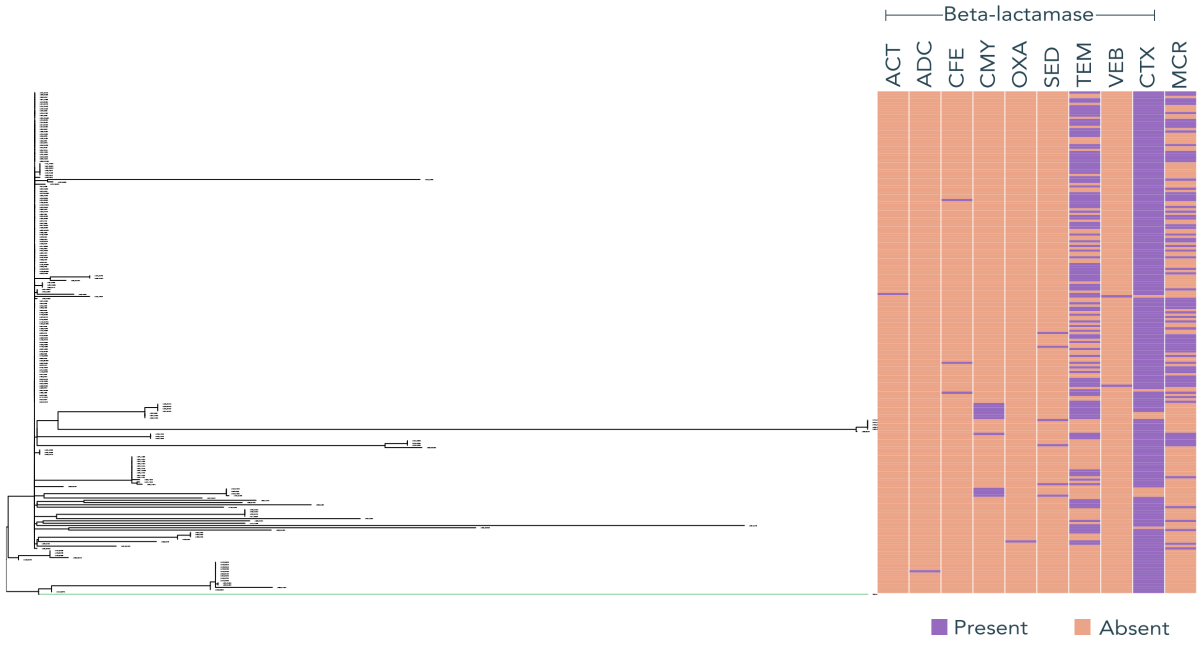

Table S3 – Total frequencies at which CTX-M subtypes were observed amongst the dataset. CTX-M-55 was the most common type observed, with other CTX-M types that are typically more dominant in other parts of the world (e.g. CTX-M-15) found to be less abundant.

CTX-M Type	Number of Isolates with CTX-M Present
CTX-M-55	64
CTX-M-14	58
CTX-M-159	57
CTX-M-15	30
CTX-M-102	25
CTX-M-40	2
CTX-M-63	2
CTX-M-164	1
CTX-M-181	1
CTX-M-196	1
CTX-M-32	1
CTX-M-65	1
CTX-M-76	1
CTX-M-77	1

Table S4 – Assembly statistics of isolates determined to be linked. Reference sequences used for mapping are displayed in bold. Isolate names encode both isolate ID (LAXXX), participant number (_XX_) and date of isolation (_XXXX).

Isolate	# contigs (>= 0 bp)	# contigs (>= 1 kb)	Total length	GC (%)	N50	Cluster
**LA069_11_2709**	**341**	**154**	**5241930**	**50.48**	**75827**	11/11B
LA070_11_2909	333	146	5230435	50.47	73956	
LA071_11_3009	317	149	5225168	50.49	75889	
LA072_11_0110	344	151	5228373	50.48	75322	
LA073_11_0210	325	145	5226866	50.48	76162	
LA074_11_0310	324	156	5220536	50.47	69588	
LA075_11_0410	338	154	5229790	50.49	73113	
LA076_11_0510	340	149	5227973	50.48	75893	
LA077_11_0610	326	140	5231763	50.49	78744	
LA078_11_0710	349	153	5227688	50.48	73113	
LA080_11_0810	342	169	5214475	50.48	70562	
LA081_11B_2809	326	152	5223801	50.49	75893	
**LA118_17_2509**	**220**	**88**	**4892186**	**50.85**	**185871**	17/06 33/05
LA121_17_0210	194	86	4890061	50.85	185871	
LA200_33_0310	258	109	5001032	50.75	144139	
LA035_6_0410	207	88	4925718	50.85	169227	
LA201_33_0510	273	102	5008310	50.76	120237	
LA028_5_0810	318	101	5034603	50.76	144138	
**LA023_5_3009**	**201**	**54**	**5040268**	**50.47**	**181899**	05/13/40
LA094_13_0210	208	58	5043170	50.46	181679	
LA095_13_0310	291	56	5083612	50.44	290488	
LA251_40_0510	391	73	5262056	50.47	181679	
LA252_40_0610	220	67	5154959	50.46	210450	
LA253_40_0710	198	64	5149247	50.47	210449	
LA254_40_0810	211	65	5152797	50.46	181105	
**LA137_19_0210**	**114**	**56**	**5023902**	**50.61**	**197781**	19/34
LA138_19_0310	114	57	5022903	50.61	193427	
LA219_34_0710	115	57	5025340	50.54	196563	
**LA033_6_2509**	**219**	**79**	**5127269**	**50.59**	**149048**	06/21
LA151_21_2709	212	86	5125423	50.48	150580	
LA153_21_2809	173	88	5040037	50.56	136467	
LA158_21_0310	271	80	5171541	50.59	148969	

Figure S3 – Virulence genes detected in *E. coli* isolates across the dataset. The majority of isolates are ExPECs, however some EPECs were detected, and belong to sequence types 48 and 40. Further details are provided in Table S5 (below).

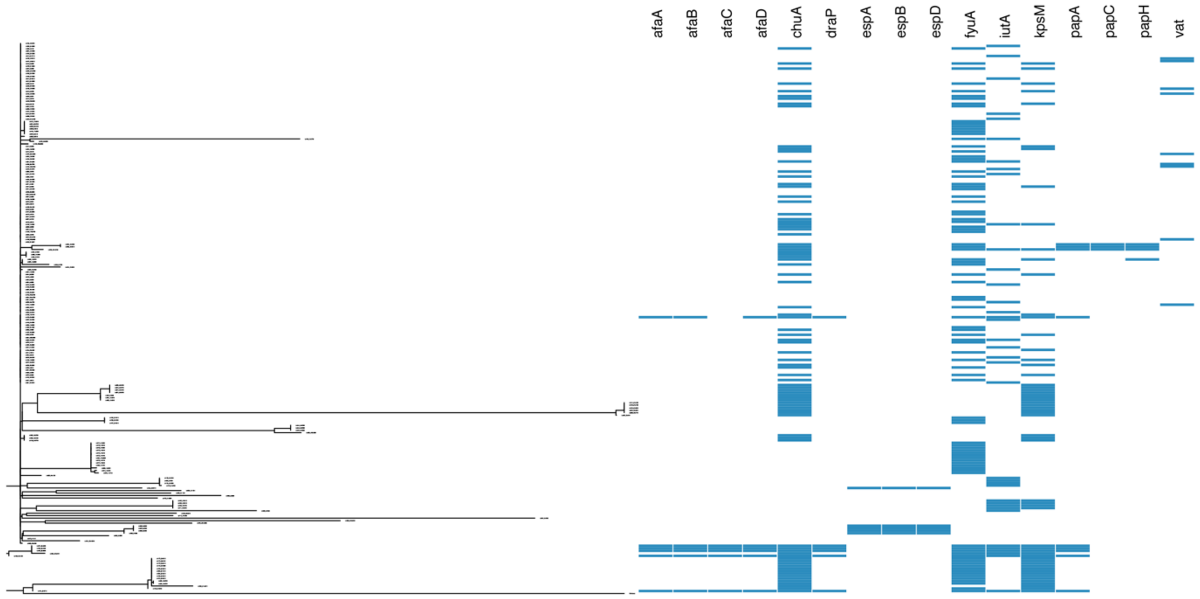

Table S5 – *E. coli* sequence types, phylogroups and virulence genes. 1 indicates presence of virulence gene, blank indicates absence.

ID	ST	Phylogroup	afaA	afaB	afaC	afaD	chuA	draP	espA	espB	espD	fyuA	iutA	kpsM	papA	papC	papH	vat
LA001_3-2309	195	A										1						
LA002_3-2509	1722	F					1					1						
LA004_3-2609	1722	F					1					1						
LA006_3-2709	1722	F					1											
LA007_3-2909	1722	F					1					1						
LA008_3-3009	1722	F					1					1						
LA009_3-0110	1722	F					1					1						
LA010_3-0210	1722	F					1					1						
LA011_3-0310	1722	F					1					1						
LA013_3-0410	1722	F					1					1						
LA015_3-0510	1722	F					1					1						
LA016_3-0710	1722	F					1					1						
LA017_3-0810	1722	F					1					1						
LA019_4-2009	101	B1																
LA020_4-2309	34	A																
LA021_5-2809	1722	F					1											
LA022_5-2909	6856	A																
LA023_5-3009	38	D					1							1				
LA024_5-0110	1722	F					1											

LA026_5-0510	542	A								
LA028_5-0810	515	B1						1		
LA030_5B-2609	542	A								
LA031_5B-2609	34	A								
LA032_5C-2907	101	B1								
LA033_6-2509	3285	B1							1	
LA035_6-0410	515	B1						1		
LA039_6B-2309	101	B1								
LA041_8-2009	69	D	1					1		1
LA042_8-2109	69	D	1					1		1
LA043_8-2209	69	D	1					1		1
LA044_8-2309	69	D	1					1		1
LA046_8-2409	195	A								
LA047_8-2509	69	D	1					1		1
LA048_8-2609	69	D	1					1		1
LA049_8-2709	746	A	1					1		
LA050_8-2709	69	D	1					1		1
LA051_8-2809	69	D	1					1		1
LA053_8-0210	6984	B1								
LA054_8-0310	6984	B1								
LA055_8-0410	69	D	1					1		1
LA058_9-2009	4682	B1								
LA059_9-2009	665	A							1	
LA061_9-2109	1072	A								
LA062_9-2209	48	A			1	1	1			
LA063_9-2209	101	B1								

LA065_9-2309	48	A			1	1	1						
LA066_9-2309	48	A			1	1	1						
LA068_9-2409	48	A			1	1	1						
LA069_11-2709	2067	B1						1					
LA070_11-2909	2067	B1						1					
LA071_11-3009	2067	B1						1					
LA072_11-0110	2067	B1						1					
LA073_11-0210	2067	B1						1					
LA074_11-0310	2067	B1						1					
LA075_11-0410	2067	B1						1					
LA076_11-0510	2067	B1						1					
LA077_11-0610	2067	B1						1					
LA078_11-0710	2067	B1						1					
LA079_11-0710	2067	B1						1					
LA080_11-0810	2067	B1						1					
LA081_11B-2809	2067	B1						1					
LA082_12-2309	394	D	1							1			
LA083_12-2509	394	D	1							1			
LA084_12-2709	69	D	1					1			1	1	1
LA085_12-2809	746	A						1					
LA086_12-2909	101	B1	1							1			
LA088_12-0310	69	D	1					1			1	1	1
LA089_12-0510	746	A	1					1		1			1
LA090_13-2409	101	B1						1					
LA091_13-2509	101	B1											
LA092_13-2609	101	B1											

LA093_13-3009	1722	F	1											
LA094_13-0210	38	D	1							1				
LA095_13-0310	38	D	1							1				
LA096_15-2409	744	A												
LA097_16-2509	101	B1												
LA098_16-2609	101	B1												
LA100_16-2809	101	B1	1					1		1				
LA101_16-2909	101	B1												
LA102_16-3009	38	D	1						1	1				
LA103_16-0110	93	A							1	1				
LA104_16-0110	101	B1												
LA105_16-0210	93	A							1	1				
LA106_16-0310	93	A							1	1				
LA108_16-0410	38	D	1							1				
LA110_16-0510	101	B1												
LA111_16-0610	93	A							1	1				
LA112_16A-2109	744	A												
LA115_17-2409	1638	A												
LA118_17-2509	515	B1						1						
LA121_17-0210	515	B1						1						
LA124_17-0310	542	A												
LA129_17B-2709	515	B1						1						
LA130_18-2709	410	C						1	1					
LA132_19-2309	34	A												
LA133_19-2609	34	A												1
LA134_19-2809	34	A												1

LA135_19-2909	101	B1																
LA136_19-3009	34	A																1
LA137_19-0210	34	A																1
LA138_19-0310	34	A																1
LA139_19B-2209	6799	B1																
LA140_19B-2709	34	A																1
LA142_21-2109	34	A					1							1				
LA144_21-2409	34	A					1							1				
LA146_21-2509	101	B1																
LA151_21-2709	3285	B1											1					
LA153_21-2809	201	B1																
LA154_21-2809	3285	B1											1					
LA155_21-3009	101	B1																
LA156_21-0110	10	A										1						
LA157_21-0210	10	A										1						
LA158_21-0310	3285	B1											1					
LA160_21-0410	10	A										1						
LA163_21-0610	34	A					1							1				
LA164_21-0710	69	D					1							1				
LA165_23-2009	5895	B2	1	1	1	1	1	1				1	1	1	1			
LA166_23-2109	5895	B2	1	1	1	1	1	1				1	1	1	1			
LA167_23-2209	5895	B2	1	1	1	1	1	1				1	1	1	1			
LA168_23-2409	5895	B2	1	1	1	1	1	1				1	1	1	1			
LA169_23-2809	34	A																
LA170_23-2909	542	A	1	1		1	1	1				1	1	1	1			
LA172_23-3009	457	F					1					1		1				

LA173_23-0110	457	F					1					1		1				
LA174_23-0210	457	F					1					1		1				
LA175_23-0310	457	F					1					1		1				
LA178_23-0410	457	F					1					1		1				
LA179_23-0610	457	F					1					1		1				
LA180_23-0610	457	F					1					1		1				
LA181_23-0710	457	F	1	1	1	1	1	1				1	1	1	1			
LA183_23-0810	457	F					1					1		1				
LA184_26-2409	34	A												1				
LA186_26-2709	34	A																1
LA187_26A-2509	34	A																1
LA188_26B-2609	34	A																1
LA189_33-1909	34	A										1						
LA191_33-2009	34	A										1						
LA192_33-2109	34	A																
LA193_33-2209	34	A																
LA194_33-2309	34	A																
LA195_33-2409	34	A										1						
LA196_33-2509	34	A																
LA197_33-2809	195	A																
LA198_33-2909	195	A																
LA199_33-0210	195	A																
LA200_33-0310	515	B1										1						
LA201_33-0510	515	B1										1						
LA202_33-0710	195	A					1							1				
LA203_33-0810	38	D					1							1				

LA204_33A-2509	515	B1		1		
LA205_34-2109	1081	B1				
LA206_34-2209	167	A			1	
LA207_34-2509	617	A			1	
LA208_34-2609	617	A			1	
LA209_34-2709	167	A			1	
LA210_34-2809	167	A			1	
LA211_34-2909	167	A			1	
LA212_34-3009	34	A				
LA213_34-0110	167	A			1	
LA214_34-0210	34	A			1	
LA215_34-0310	34	A			1	
LA216_34-0410	34	A			1	
LA217_34-0510	167	A			1	
LA218_34-0610	167	A			1	
LA219_34-0710	34	A			1	
LA220_34-0810	34	A			1	
LA221_34-0910	34	A			1	
LA222_34A-2309	167	A			1	
LA223_34B-2409	195	A				
LA224_35-2009	167	A	1	1	1	
LA227_35-2109	5848	B1				
LA228_35-2209	167	A	1	1	1	
LA230_35-2309	101	B1	1			1
LA232_35-2609	101	B1				
LA233_35A-2709	101	B1				

LA234_35B-2509	101	B1											
LA236_36-2809	101	B1											
LA237_36-3009	101	B1											
LA238_36-0210	398	A											
LA239_40-2209	195	A											
LA240_40-2309	195	A											
LA241_40-2409	195	A						1					
LA243_40-2809	48	A											
LA244_40-2909	48	A											
LA245_40-3009	48	A											
LA247_40-0210	101	B1											
LA249_40-0410	40	B1			1	1	1						
LA251_40-0510	38	D	1							1			
LA252_40-0610	38	D	1							1			
LA253_40-0710	38	D	1							1			
LA254_40-0810	38	D	1							1			
LA255_40A-2509	206	A											
LA256_40A-2509	195	A											
LA258_40B-2809	34	A											
LA259_40B-0310	5870	A											
LA260_40C-2709	69	D	1					1	1	1	1	1	1
LA261_3-1910	1722	F	1					1					
LA262_3-2510	1722	F	1					1					
LA263_3-0211	1722	F	1					1					
LA264_3-1811	1722	F	1					1					
LA266_3-0912	1722	F	1					1					

LA267_3-1512	1722	F	1	1		
LA268_3-2112	1722	F	1	1		
LA269_3-0601	1722	F	1	1		
LA270_3-1210	1722	F	1	1		
LA271_3-1901	1722	F	1	1		
LA272_3-2501	1722	F	1	1		
LA273_8-1310	195	A				
LA275_8-2909	69	D	1	1		1
LA294_16-2610	457	F	1	1		1
LA295_16-2910	457	F	1	1		1
LA296_16-1301	224	B1				
LA300_19B-2111	131	B2	1	1	1	1
LA301_23A-0810	48	A				
LA302_21A-0311	457	F	1			1
LA303_23-1210	457	F	1	1		1
LA304_23-2010	457	F	1	1		1
LA305_33-0111	457	D	1			1
LA306_34-1810	224	B1				

Table S6 – Isolate metadata, including participant number, isolation date, species and accession numbers.

ID	Participant	Date	Organism	Biosample Accession	Experiment Accession	SRA Accession
LA001	3	23/09/2015	*Escherichia coli*	SAMN12599825	SRX6744448	SRS5295240
LA002	3	25/09/2015	*Escherichia coli*	SAMN12599826	SRX6744447	SRS5295239
LA003	3	26/09/2015	*Citrobacter sp.*	SAMN12599827	SRX6744450	SRS5295242
LA004	3	26/09/2015	*Escherichia coli*	SAMN12599828	SRX6744449	SRS5295241
LA005	3	27/09/2015	*Citrobacter sp.*	SAMN12599829	SRX6744452	SRS5295244
LA006	3	27/09/2015	*Escherichia coli*	SAMN12599830	SRX6744451	SRS5295243
LA007	3	29/09/2015	*Escherichia coli*	SAMN12599831	SRX6744454	SRS5295246
LA008	3	30/09/2015	*Escherichia coli*	SAMN12599832	SRX6744453	SRS5295245
LA009	3	01/10/2015	*Escherichia coli*	SAMN12599833	SRX6744446	SRS5295238
LA010	3	02/10/2015	*Escherichia coli*	SAMN12599834	SRX6744445	SRS5295237
LA011	3	03/10/2015	*Escherichia coli*	SAMN12599835	SRX6744262	SRS5295054
LA012	3	04/10/2015	*Citrobacter sp.*	SAMN12599836	SRX6744261	SRS5295053
LA013	3	04/10/2015	*Escherichia coli*	SAMN12599837	SRX6744264	SRS5295056
LA014	3	05/10/2015	*Klebsiella pneumoniae*	SAMN12599838	SRX6744263	SRS5295055
LA015	3	05/10/2015	*Escherichia coli*	SAMN12599839	SRX6744266	SRS5295058
LA016	3	07/10/2015	*Escherichia coli*	SAMN12599840	SRX6744265	SRS5295057
LA017	3	08/10/2015	*Escherichia coli*	SAMN12599841	SRX6744268	SRS5295060
LA018	4	20/09/2015	*Klebsiella pneumoniae*	SAMN12599842	SRX6744267	SRS5295059
LA019	4	20/09/2015	*Escherichia coli*	SAMN12599843	SRX6744270	SRS5295062
LA020	4	23/09/2015	*Escherichia coli*	SAMN12599844	SRX6744269	SRS5295061
LA021	5	28/09/2015	*Escherichia coli*	SAMN12599845	SRX6744374	SRS5295166
LA022	5	29/09/2015	*Escherichia coli*	SAMN12599846	SRX6744375	SRS5295167

LA023	5	30/09/2015	*Escherichia coli*	SAMN12599847	SRX6744376	SRS5295168
LA024	5	01/10/2015	*Escherichia coli*	SAMN12599848	SRX6744377	SRS5295169
LA025	5	04/10/2015	*Klebsiella pneumoniae*	SAMN12599849	SRX6744378	SRS5295170
LA026	5	05/10/2015	*Escherichia coli*	SAMN12599850	SRX6744379	SRS5295171
LA027	5	07/10/2015	*Citrobacter sp.*	SAMN12599851	SRX6744380	SRS5295172
LA028	5	08/10/2015	*Escherichia coli*	SAMN12599852	SRX6744381	SRS5295173
LA029	5	09/10/2015	*Enterobacter cloacae*	SAMN12599853	SRX6744371	SRS5295163
LA030	5B	26/09/2015	*Escherichia coli*	SAMN12599854	SRX6744372	SRS5295164
LA031	5B	26/09/2015	*Escherichia coli*	SAMN12599855	SRX6744342	SRS5295134
LA032	5C	29/07/2015	*Escherichia coli*	SAMN12599856	SRX6744341	SRS5295133
LA033	6	25/09/2015	*Escherichia coli*	SAMN12599857	SRX6744340	SRS5295132
LA034	6	29/09/2015	*Enterobacter cloacae*	SAMN12599858	SRX6744339	SRS5295131
LA035	6	04/10/2015	*Escherichia coli*	SAMN12599859	SRX6744338	SRS5295130
LA036	6A	23/09/2015	*Citrobacter sp.*	SAMN12599860	SRX6744337	SRS5295129
LA038	6B	23/09/2015	*Klebsiella pneumoniae*	SAMN12599861	SRX6744336	SRS5295128
LA039	6B	23/09/2015	*Escherichia coli*	SAMN12599862	SRX6744335	SRS5295127
LA040	7	23/09/2015	*Enterobacter cloacae*	SAMN12599863	SRX6744401	SRS5295193
LA041	8	20/09/2015	*Escherichia coli*	SAMN12599864	SRX6744333	SRS5295125
LA042	8	21/09/2015	*Escherichia coli*	SAMN12599865	SRX6744443	SRS5295235
LA043	8	22/09/2015	*Escherichia coli*	SAMN12599866	SRX6744444	SRS5295236
LA044	8	23/09/2015	*Escherichia coli*	SAMN12599867	SRX6744441	SRS5295233
LA045	8	24/09/2015	*Enterobacter cloacae*	SAMN12599868	SRX6744442	SRS5295234
LA046	8	24/09/2015	*Escherichia coli*	SAMN12599869	SRX6744439	SRS5295231
LA047	8	25/09/2015	*Escherichia coli*	SAMN12599870	SRX6744440	SRS5295232
LA048	8	26/09/2015	*Escherichia coli*	SAMN12599871	SRX6744437	SRS5295229
LA049	8	27/09/2015	*Escherichia coli*	SAMN12599872	SRX6744438	SRS5295230

LA050	8	27/09/2015	*Escherichia coli*	SAMN12599873	SRX6744435	SRS5295227
LA051	8	28/09/2015	*Escherichia coli*	SAMN12599874	SRX6744436	SRS5295228
LA052	8	29/09/2015	*Klebsiella pneumoniae*	SAMN12599875	SRX6744412	SRS5295204
LA053	8	02/10/2015	*Escherichia coli*	SAMN12599876	SRX6744411	SRS5295203
LA054	8	03/10/2015	*Escherichia coli*	SAMN12599877	SRX6744313	SRS5295105
LA055	8	04/10/2015	*Escherichia coli*	SAMN12599878	SRX6744413	SRS5295205
LA056	9	20/09/2015	*Klebsiella pneumoniae*	SAMN12599879	SRX6744330	SRS5295122
LA057	9	20/09/2015	*Enterobacter cloacae*	SAMN12599880	SRX6744407	SRS5295199
LA058	9	20/09/2015	*Escherichia coli*	SAMN12599881	SRX6744410	SRS5295202
LA059	9	20/09/2015	*Escherichia coli*	SAMN12599882	SRX6744409	SRS5295201
LA060	9	21/09/2015	*Klebsiella pneumoniae*	SAMN12599883	SRX6744406	SRS5295198
LA061	9	21/09/2015	*Escherichia coli*	SAMN12599884	SRX6744405	SRS5295197
LA062	9	22/09/2015	*Escherichia coli*	SAMN12599885	SRX6744218	SRS5295010
LA063	9	22/09/2015	*Escherichia coli*	SAMN12599886	SRX6744383	SRS5295175
LA064	9	22/09/2015	*Escherichia coli*	SAMN12599887	SRX6744220	SRS5295012
LA065	9	23/09/2015	*Escherichia coli*	SAMN12599888	SRX6744221	SRS5295013
LA066	9	23/09/2015	*Escherichia coli*	SAMN12599889	SRX6744274	SRS5295066
LA067	9	23/09/2015	*Citrobacter sp.*	SAMN12599890	SRX6744215	SRS5295007
LA068	9	24/09/2015	*Escherichia coli*	SAMN12599891	SRX6744216	SRS5295008
LA069	11	27/09/2015	*Escherichia coli*	SAMN12599892	SRX6744217	SRS5295009
LA070	11	29/09/2015	*Escherichia coli*	SAMN12599893	SRX6744223	SRS5295015
LA071	11	30/09/2015	*Escherichia coli*	SAMN12599894	SRX6744403	SRS5295195
LA072	11	01/10/2015	*Escherichia coli*	SAMN12599895	SRX6744478	SRS5295270
LA073	11	02/10/2015	*Escherichia coli*	SAMN12599896	SRX6744477	SRS5295269
LA074	11	03/10/2015	*Escherichia coli*	SAMN12599897	SRX6744476	SRS5295268
LA075	11	04/10/2015	*Escherichia coli*	SAMN12599898	SRX6744475	SRS5295267

LA076	11	05/10/2015	*Escherichia coli*	SAMN12599899	SRX6744482	SRS5295274
LA077	11	06/10/2015	*Escherichia coli*	SAMN12599900	SRX6744481	SRS5295273
LA078	11	07/10/2015	*Escherichia coli*	SAMN12599901	SRX6744480	SRS5295272
LA079	11	07/10/2015	*Escherichia coli*	SAMN12599902	SRX6744479	SRS5295271
LA080	11	08/10/2015	*Escherichia coli*	SAMN12599903	SRX6744474	SRS5295266
LA081	11B	28/09/2015	*Escherichia coli*	SAMN12599904	SRX6744433	SRS5295225
LA082	12	23/09/2015	*Escherichia coli*	SAMN12599905	SRX6744293	SRS5295085
LA083	12	25/09/2015	*Escherichia coli*	SAMN12599906	SRX6744294	SRS5295086
LA084	12	27/09/2015	*Escherichia coli*	SAMN12599907	SRX6744400	SRS5295192
LA085	12	28/09/2015	*Escherichia coli*	SAMN12599908	SRX6744382	SRS5295174
LA086	12	29/09/2015	*Escherichia coli*	SAMN12599909	SRX6744297	SRS5295089
LA088	12	03/10/2015	*Escherichia coli*	SAMN12599910	SRX6744310	SRS5295102
LA089	12	05/10/2015	*Escherichia coli*	SAMN12599911	SRX6744295	SRS5295087
LA090	13	24/09/2015	*Escherichia coli*	SAMN12599912	SRX6744296	SRS5295088
LA091	13	25/09/2015	*Escherichia coli*	SAMN12599913	SRX6744402	SRS5295194
LA092	13	26/09/2015	*Escherichia coli*	SAMN12599914	SRX6744404	SRS5295196
LA093	13	30/09/2015	*Escherichia coli*	SAMN12599915	SRX6744244	SRS5295036
LA094	13	02/10/2015	*Escherichia coli*	SAMN12599916	SRX6744243	SRS5295035
LA095	13	03/10/2015	*Escherichia coli*	SAMN12599917	SRX6744213	SRS5295005
LA096	15	24/09/2015	*Escherichia coli*	SAMN12599918	SRX6744210	SRS5295002
LA097	16	25/09/2015	*Escherichia coli*	SAMN12599919	SRX6744248	SRS5295040
LA098	16	26/09/2015	*Escherichia coli*	SAMN12599920	SRX6744247	SRS5295039
LA099	16	27/09/2015	*Enterobacter cloacae*	SAMN12599921	SRX6744250	SRS5295042
LA100	16	28/09/2015	*Escherichia coli*	SAMN12599922	SRX6744249	SRS5295041
LA101	16	29/09/2015	*Escherichia coli*	SAMN12599923	SRX6744472	SRS5295264
LA102	16	30/09/2015	*Escherichia coli*	SAMN12599924	SRX6744471	SRS5295263

LA103	16	01/10/2015	*Escherichia coli*	SAMN12599925	SRX6744229	SRS5295021
LA104	16	01/10/2015	*Escherichia coli*	SAMN12599926	SRX6744230	SRS5295022
LA105	16	02/10/2015	*Escherichia coli*	SAMN12599927	SRX6744227	SRS5295019
LA106	16	03/10/2015	*Escherichia coli*	SAMN12599928	SRX6744228	SRS5295020
LA107	16	03/10/2015	*Citrobacter sp.*	SAMN12599929	SRX6744233	SRS5295025
LA108	16	04/10/2015	*Escherichia coli*	SAMN12599930	SRX6744234	SRS5295026
LA109	16	04/10/2015	*Citrobacter sp.*	SAMN12599931	SRX6744231	SRS5295023
LA110	16	05/10/2015	*Escherichia coli*	SAMN12599932	SRX6744232	SRS5295024
LA111	16	06/10/2015	*Escherichia coli*	SAMN12599933	SRX6744225	SRS5295017
LA112	16A	21/09/2015	*Escherichia coli*	SAMN12599934	SRX6744226	SRS5295018
LA113	16A	21/09/2015	*Citrobacter sp.*	SAMN12599935	SRX6744276	SRS5295068
LA114	16B	21/09/2015	*Citrobacter sp.*	SAMN12599936	SRX6744275	SRS5295067
LA115	17	24/09/2015	*Escherichia coli*	SAMN12599937	SRX6744278	SRS5295070
LA116	17	24/09/2015	*Enterobacter cloacae*	SAMN12599938	SRX6744277	SRS5295069
LA117	17	25/09/2015	*Citrobacter sp.*	SAMN12599939	SRX6744280	SRS5295072
LA118	17	25/09/2015	*Escherichia coli*	SAMN12599940	SRX6744279	SRS5295071
LA119	17	29/09/2015	*Citrobacter sp.*	SAMN12599941	SRX6744282	SRS5295074
LA120	17	30/09/2015	*Citrobacter sp.*	SAMN12599942	SRX6744281	SRS5295073
LA121	17	02/10/2015	*Escherichia coli*	SAMN12599943	SRX6744273	SRS5295065
LA122	17	02/10/2015	*Citrobacter sp.*	SAMN12599944	SRX6744272	SRS5295064
LA123	17	03/10/2015	*Klebsiella pneumoniae*	SAMN12599945	SRX6744318	SRS5295110
LA124	17	03/10/2015	*Escherichia coli*	SAMN12599946	SRX6744319	SRS5295111
LA125	17	04/10/2015	*Citrobacter sp.*	SAMN12599947	SRX6744320	SRS5295112
LA126	17	04/10/2015	*Citrobacter sp.*	SAMN12599948	SRX6744321	SRS5295113
LA127	17A	26/09/2015	*Citrobacter sp.*	SAMN12599949	SRX6744314	SRS5295106
LA128	17B	27/09/2015	*Citrobacter sp.*	SAMN12599950	SRX6744315	SRS5295107

LA129	17B	27/09/2015	*Escherichia coli*	SAMN12599951	SRX6744316	SRS5295108
LA130	18	27/09/2015	*Escherichia coli*	SAMN12599952	SRX6744317	SRS5295109
LA132	19	23/09/2015	*Escherichia coli*	SAMN12599953	SRX6744326	SRS5295118
LA133	19	26/09/2015	*Escherichia coli*	SAMN12599954	SRX6744327	SRS5295119
LA134	19	28/09/2015	*Escherichia coli*	SAMN12599955	SRX6744356	SRS5295148
LA135	19	29/09/2015	*Escherichia coli*	SAMN12599956	SRX6744355	SRS5295147
LA136	19	30/09/2015	*Escherichia coli*	SAMN12599957	SRX6744354	SRS5295146
LA137	19	02/10/2015	*Escherichia coli*	SAMN12599958	SRX6744353	SRS5295145
LA138	19	03/10/2015	*Escherichia coli*	SAMN12599959	SRX6744360	SRS5295152
LA139	19B	22/09/2015	*Escherichia coli*	SAMN12599960	SRX6744359	SRS5295151
LA140	19B	27/09/2015	*Escherichia coli*	SAMN12599961	SRX6744358	SRS5295150
LA141	21	18/09/2015	*Stenotrophomonas maltophilia*	SAMN12599962	SRX6744357	SRS5295149
LA142	21	21/09/2015	*Escherichia coli*	SAMN12599963	SRX6744364	SRS5295156
LA143	21	21/09/2015	*Citrobacter sp.*	SAMN12599964	SRX6744363	SRS5295155
LA144	21	24/09/2015	*Escherichia coli*	SAMN12599965	SRX6744390	SRS5295182
LA145	21	25/09/2015	*Citrobacter sp.*	SAMN12599966	SRX6744391	SRS5295183
LA146	21	25/09/2015	*Escherichia coli*	SAMN12599967	SRX6744388	SRS5295180
LA147	21	26/09/2015	*Citrobacter sp.*	SAMN12599968	SRX6744389	SRS5295181
LA148	21	26/09/2015	*Citrobacter sp.*	SAMN12599969	SRX6744386	SRS5295178
LA149	11	31/12/2015	*Acinetobacter sp.*	SAMN12599970	SRX6744387	SRS5295179
LA150	21	27/09/2015	*Citrobacter sp.*	SAMN12599971	SRX6744384	SRS5295176
LA151	21	27/09/2015	*Escherichia coli*	SAMN12599972	SRX6744385	SRS5295177
LA152	21	28/09/2015	*Klebsiella pneumoniae*	SAMN12599973	SRX6744398	SRS5295190
LA153	21	28/09/2015	*Escherichia coli*	SAMN12599974	SRX6744399	SRS5295191
LA154	21	28/09/2015	*Escherichia coli*	SAMN12599975	SRX6744421	SRS5295213

LA155	21	30/09/2015	*Escherichia coli*	SAMN12599976	SRX6744420	SRS5295212
LA156	21	01/10/2015	*Escherichia coli*	SAMN12599977	SRX6744423	SRS5295215
LA157	21	02/10/2015	*Escherichia coli*	SAMN12599978	SRX6744422	SRS5295214
LA158	21	03/10/2015	*Escherichia coli*	SAMN12599979	SRX6744417	SRS5295209
LA159	21	04/10/2015	*Citrobacter sp.*	SAMN12599980	SRX6744416	SRS5295208
LA160	21	04/10/2015	*Escherichia coli*	SAMN12599981	SRX6744419	SRS5295211
LA161	21	05/10/2015	*Enterobacter cloacae*	SAMN12599982	SRX6744418	SRS5295210
LA162	21	05/10/2015	*Klebsiella pneumoniae*	SAMN12599983	SRX6744429	SRS5295221
LA163	21	06/10/2015	*Escherichia coli*	SAMN12599984	SRX6744428	SRS5295220
LA164	21	07/10/2015	*Escherichia coli*	SAMN12599985	SRX6744455	SRS5295247
LA165	23	20/09/2015	*Escherichia coli*	SAMN12599986	SRX6744456	SRS5295248
LA166	23	21/09/2015	*Escherichia coli*	SAMN12599987	SRX6744457	SRS5295249
LA167	23	22/09/2015	*Escherichia coli*	SAMN12599988	SRX6744458	SRS5295250
LA168	23	24/09/2015	*Escherichia coli*	SAMN12599989	SRX6744459	SRS5295251
LA169	23	28/09/2015	*Escherichia coli*	SAMN12599990	SRX6744460	SRS5295252
LA170	23	29/09/2015	*Escherichia coli*	SAMN12599991	SRX6744461	SRS5295253
LA171	23	30/09/2015	*Klebsiella pneumoniae*	SAMN12599992	SRX6744462	SRS5295254
LA172	23	30/09/2015	*Escherichia coli*	SAMN12599993	SRX6744463	SRS5295255
LA173	23	01/10/2015	*Escherichia coli*	SAMN12599994	SRX6744464	SRS5295256
LA174	23	02/10/2015	*Escherichia coli*	SAMN12599995	SRX6744201	SRS5294993
LA175	23	03/10/2015	*Escherichia coli*	SAMN12599996	SRX6744200	SRS5294992
LA176	23	04/10/2015	*Enterobacter cloacae*	SAMN12599997	SRX6744199	SRS5294991
LA177	23	04/10/2015	*Enterobacter cloacae*	SAMN12599998	SRX6744198	SRS5294990
LA178	23	04/10/2015	*Escherichia coli*	SAMN12599999	SRX6744197	SRS5294989
LA179	23	06/10/2015	*Escherichia coli*	SAMN12600000	SRX6744196	SRS5294988
LA180	23	06/10/2015	*Escherichia coli*	SAMN12600001	SRX6744195	SRS5294987

LA181	23	07/10/2015	*Escherichia coli*	SAMN12600002	SRX6744194	SRS5294986
LA182	23	08/10/2015	*Klebsiella pneumoniae*	SAMN12600003	SRX6744193	SRS5294985
LA183	23	08/10/2015	*Escherichia coli*	SAMN12600004	SRX6744192	SRS5294984
LA184	26	24/09/2015	*Escherichia coli*	SAMN12600005	SRX6744253	SRS5295045
LA186	26	27/09/2015	*Escherichia coli*	SAMN12600006	SRX6744254	SRS5295046
LA187	26A	25/09/2015	*Escherichia coli*	SAMN12600007	SRX6744251	SRS5295043
LA188	26B	26/09/2015	*Escherichia coli*	SAMN12600008	SRX6744252	SRS5295044
LA189	33	19/09/2015	*Escherichia coli*	SAMN12600009	SRX6744257	SRS5295049
LA191	33	20/09/2015	*Escherichia coli*	SAMN12600010	SRX6744258	SRS5295050
LA192	33	21/09/2015	*Escherichia coli*	SAMN12600011	SRX6744255	SRS5295047
LA193	33	22/09/2015	*Escherichia coli*	SAMN12600012	SRX6744256	SRS5295048
LA194	33	23/09/2015	*Escherichia coli*	SAMN12600013	SRX6744259	SRS5295051
LA195	33	24/09/2015	*Escherichia coli*	SAMN12600014	SRX6744260	SRS5295052
LA196	33	25/09/2015	*Escherichia coli*	SAMN12600015	SRX6744300	SRS5295092
LA197	33	28/09/2015	*Escherichia coli*	SAMN12600016	SRX6744299	SRS5295091
LA198	33	29/09/2015	*Escherichia coli*	SAMN12600017	SRX6744302	SRS5295094
LA199	33	02/10/2015	*Escherichia coli*	SAMN12600018	SRX6744301	SRS5295093
LA200	33	03/10/2015	*Escherichia coli*	SAMN12600019	SRX6744304	SRS5295096
LA201	33	05/10/2015	*Escherichia coli*	SAMN12600020	SRX6744303	SRS5295095
LA202	33	07/10/2015	*Escherichia coli*	SAMN12600021	SRX6744306	SRS5295098
LA203	33	08/10/2015	*Escherichia coli*	SAMN12600022	SRX6744305	SRS5295097
LA204	33A	25/09/2015	*Escherichia coli*	SAMN12600023	SRX6744308	SRS5295100
LA205	34	21/09/2015	*Escherichia coli*	SAMN12600024	SRX6744307	SRS5295099
LA206	34	22/09/2015	*Escherichia coli*	SAMN12600025	SRX6744284	SRS5295076
LA207	34	25/09/2015	*Escherichia coli*	SAMN12600026	SRX6744283	SRS5295075
LA208	34	26/09/2015	*Escherichia coli*	SAMN12600027	SRX6744286	SRS5295078

LA209	34	27/09/2015	*Escherichia coli*	SAMN12600028	SRX6744285	SRS5295077
LA210	34	28/09/2015	*Escherichia coli*	SAMN12600029	SRX6744288	SRS5295080
LA211	34	29/09/2015	*Escherichia coli*	SAMN12600030	SRX6744287	SRS5295079
LA212	34	30/09/2015	*Escherichia coli*	SAMN12600031	SRX6744290	SRS5295082
LA213	34	01/10/2015	*Escherichia coli*	SAMN12600032	SRX6744289	SRS5295081
LA214	34	02/10/2015	*Escherichia coli*	SAMN12600033	SRX6744292	SRS5295084
LA215	34	03/10/2015	*Escherichia coli*	SAMN12600034	SRX6744291	SRS5295083
LA216	34	04/10/2015	*Escherichia coli*	SAMN12600035	SRX6744237	SRS5295029
LA217	34	05/10/2015	*Escherichia coli*	SAMN12600036	SRX6744238	SRS5295030
LA218	34	06/10/2015	*Escherichia coli*	SAMN12600037	SRX6744235	SRS5295027
LA219	34	07/10/2015	*Escherichia coli*	SAMN12600038	SRX6744236	SRS5295028
LA220	34	08/10/2015	*Escherichia coli*	SAMN12600039	SRX6744241	SRS5295033
LA221	34	09/10/2015	*Escherichia coli*	SAMN12600040	SRX6744242	SRS5295034
LA222	34A	23/09/2015	*Escherichia coli*	SAMN12600041	SRX6744239	SRS5295031
LA223	34B	24/09/2015	*Escherichia coli*	SAMN12600042	SRX6744240	SRS5295032
LA224	35	20/09/2015	*Escherichia coli*	SAMN12600043	SRX6744245	SRS5295037
LA225	35	20/09/2015	*Citrobacter sp.*	SAMN12600044	SRX6744246	SRS5295038
LA226	35	21/09/2015	*Citrobacter sp.*	SAMN12600045	SRX6744415	SRS5295207
LA227	35	21/09/2015	*Escherichia coli*	SAMN12600046	SRX6744224	SRS5295016
LA228	35	22/09/2015	*Escherichia coli*	SAMN12600047	SRX6744362	SRS5295154
LA229	35	22/09/2015	*Citrobacter sp.*	SAMN12600048	SRX6744361	SRS5295153
LA230	35	23/09/2015	*Escherichia coli*	SAMN12600049	SRX6744368	SRS5295160
LA232	35	26/09/2015	*Escherichia coli*	SAMN12600050	SRX6744367	SRS5295159
LA233	35A	27/09/2015	*Escherichia coli*	SAMN12600051	SRX6744366	SRS5295158
LA234	35B	25/09/2015	*Escherichia coli*	SAMN12600052	SRX6744365	SRS5295157
LA235	36	25/09/2015	*Citrobacter sp.*	SAMN12600053	SRX6744205	SRS5294997

LA236	36	28/09/2015	*Escherichia coli*	SAMN12600054	SRX6744271	SRS5295063
LA237	36	30/09/2015	*Escherichia coli*	SAMN12600055	SRX6744323	SRS5295115
LA238	36	02/10/2015	*Escherichia coli*	SAMN12600056	SRX6744329	SRS5295121
LA239	40	22/09/2015	*Escherichia coli*	SAMN12600057	SRX6744328	SRS5295120
LA240	40	23/09/2015	*Escherichia coli*	SAMN12600058	SRX6744331	SRS5295123
LA241	40	24/09/2015	*Escherichia coli*	SAMN12600059	SRX6744322	SRS5295114
LA242	40	28/09/2015	*Klebsiella pneumoniae*	SAMN12600060	SRX6744309	SRS5295101
LA243	40	28/09/2015	*Escherichia coli*	SAMN12600061	SRX6744324	SRS5295116
LA244	40	29/09/2015	*Escherichia coli*	SAMN12600062	SRX6744325	SRS5295117
LA245	40	30/09/2015	*Escherichia coli*	SAMN12600063	SRX6744373	SRS5295165
LA246	40A	03/10/2015	*Aeromonas sp.*	SAMN12600064	SRX6744298	SRS5295090
LA247	40	02/10/2015	*Escherichia coli*	SAMN12600065	SRX6744434	SRS5295226
LA249	40	04/10/2015	*Escherichia coli*	SAMN12600066	SRX6744432	SRS5295224
LA251	40	05/10/2015	*Escherichia coli*	SAMN12600067	SRX6744431	SRS5295223
LA252	40	06/10/2015	*Escherichia coli*	SAMN12600068	SRX6744430	SRS5295222
LA253	40	07/10/2015	*Escherichia coli*	SAMN12600069	SRX6744425	SRS5295217
LA254	40	08/10/2015	*Escherichia coli*	SAMN12600070	SRX6744424	SRS5295216
LA255	40A	25/09/2015	*Escherichia coli*	SAMN12600071	SRX6744427	SRS5295219
LA256	40A	25/09/2015	*Escherichia coli*	SAMN12600072	SRX6744426	SRS5295218
LA257	40B	26/09/2015	*Enterobacter cloacae*	SAMN12600073	SRX6744414	SRS5295206
LA258	40B	28/09/2015	*Escherichia coli*	SAMN12600074	SRX6744408	SRS5295200
LA259	40B	03/10/2015	*Escherichia coli*	SAMN12600075	SRX6744219	SRS5295011
LA260	40C	27/09/2015	*Escherichia coli*	SAMN12600076	SRX6744222	SRS5295014
LA261	3	19/10/2015	*Escherichia coli*	SAMN12600077	SRX6744396	SRS5295188
LA262	3	25/10/2015	*Escherichia coli*	SAMN12600078	SRX6744397	SRS5295189
LA263	3	02/11/2015	*Escherichia coli*	SAMN12600079	SRX6744394	SRS5295186

LA264	3	18/11/2015	*Escherichia coli*	SAMN12600080	SRX6744395	SRS5295187
LA265	23	02/10/2015	*Acinetobacter sp.*	SAMN12600081	SRX6744392	SRS5295184
LA266	3	09/12/2015	*Escherichia coli*	SAMN12600082	SRX6744393	SRS5295185
LA267	3	15/12/2015	*Escherichia coli*	SAMN12600083	SRX6744191	SRS5294983
LA268	3	21/12/2015	*Escherichia coli*	SAMN12600084	SRX6744473	SRS5295265
LA269	3	06/01/2015	*Escherichia coli*	SAMN12600085	SRX6744209	SRS5295001
LA270	3	12/11/2015	*Escherichia coli*	SAMN12600086	SRX6744208	SRS5295000
LA271	3	19/01/2015	*Escherichia coli*	SAMN12600087	SRX6744207	SRS5294999
LA272	3	25/01/2015	*Escherichia coli*	SAMN12600088	SRX6744206	SRS5294998
LA273	8	13/10/2015	*Escherichia coli*	SAMN12600089	SRX6744214	SRS5295006
LA274	21A	18/09/2015	*Acinetobacter sp.*	SAMN12600090	SRX6744204	SRS5294996
LA275	8	29/09/2015	*Escherichia coli*	SAMN12600091	SRX6744203	SRS5294995
LA276	9	20/09/2015	*Klebsiella pneumoniae*	SAMN12600092	SRX6744202	SRS5294994
LA277	11	11/10/2015	*Klebsiella pneumoniae*	SAMN12600093	SRX6744212	SRS5295004
LA278	11	18/10/2015	*Klebsiella pneumoniae*	SAMN12600094	SRX6744211	SRS5295003
LA279	11	29/11/2015	*Acinetobacter sp.*	SAMN12600095	SRX6744369	SRS5295161
LA280	11	07/12/2015	*Acinetobacter sp.*	SAMN12600096	SRX6744370	SRS5295162
LA281	11	13/12/2015	*Stenotrophomonas maltophilia*	SAMN12600097	SRX6744465	SRS5295257
LA283	11	27/12/2015	*Acinetobacter sp.*	SAMN12600098	SRX6744466	SRS5295258
LA285	11B	01/11/2015	*Acinetobacter sp.*	SAMN12600099	SRX6744467	SRS5295259
LA288	11B	29/11/2015	*Acinetobacter sp.*	SAMN12600100	SRX6744468	SRS5295260
LA289	11B	07/12/2015	*Acinetobacter sp.*	SAMN12600101	SRX6744469	SRS5295261
LA290	11B	13/12/2015	*Stenotrophomonas maltophilia*	SAMN12600102	SRX6744470	SRS5295262
LA291	11B	20/12/2015	*Acinetobacter sp.*	SAMN12600103	SRX6744332	SRS5295124

LA292	11B	27/12/2015	*Acinetobacter sp.*	SAMN12600104	SRX6744334	SRS5295126
LA293	11B	31/12/2015	*Acinetobacter sp.*	SAMN12600105	SRX6744346	SRS5295138
LA294	16	26/10/2015	*Escherichia coli*	SAMN12600106	SRX6744345	SRS5295137
LA295	16	29/10/2015	*Escherichia coli*	SAMN12600107	SRX6744348	SRS5295140
LA296	16	13/01/2015	*Escherichia coli*	SAMN12600108	SRX6744347	SRS5295139
LA298	19	08/12/2015	*Achromobacter sp.*	SAMN12600109	SRX6744350	SRS5295142
LA300	19B	21/11/2015	*Escherichia coli*	SAMN12600110	SRX6744349	SRS5295141
LA301	23A	08/10/2015	*Escherichia coli*	SAMN12600111	SRX6744352	SRS5295144
LA302	21A	03/11/2015	*Escherichia coli*	SAMN12600112	SRX6744351	SRS5295143
LA303	23	12/10/2015	*Escherichia coli*	SAMN12600113	SRX6744344	SRS5295136
LA304	23	20/10/2015	*Escherichia coli*	SAMN12600114	SRX6744343	SRS5295135
LA305	33	01/11/2015	*Escherichia coli*	SAMN12600115	SRX6744311	SRS5295103
LA306	34	18/10/2015	*Escherichia coli*	SAMN12600116	SRX6744312	SRS5295104
